# A novel method of cultivating cardiac myocytes in agarose microchamber chips for studying cell synchronization

**DOI:** 10.1186/1477-3155-2-9

**Published:** 2004-09-09

**Authors:** Kensuke Kojima, Tomoyuki Kaneko, Kenji Yasuda

**Affiliations:** 1 Department of Life Sciences, Graduate school of Arts and Sciences, University of Tokyo, 3-8-1 Komaba, Meguro, Tokyo 153-8902 JAPAN

## Abstract

We have developed a new method that enables agar microstructures to be used to cultivate cardiac myocyte cells in a manner that allows their connection patterns to be controlled. Non-contact three-dimensional photo-thermal etching with a 1064-nm infrared focused laser beam was used to form the shapes of agar microstructures. This wavelength was selected as it is not absorbed by water or agar. Identical rat cardiac myocytes were cultured in adjacent microstructures connected by microchannels and the interactions of asynchronous beating cardiac myocyte cells observed. Two isolated and independently beating cardiac myocytes were shown to form contacts through the narrow microchannels and by 90 minutes had synchronized their oscillations. This occurred by one of the two cells stopping their oscillation and following the pattern of the other cell. In contrast, when two sets of synchronized beating cells came into contact, those two sets synchronized without any observable interruptions to their rhythms. The results indicate that the synchronization process of cardiac myocytes may be dependent on the community size and network pattern of these cells.

## Finding

Single-cell based analysis methods have become more and more important for understanding the cell-group effects such as how information is controlled and recorded in a cell group or a network shape. Early tissue culture studies of cardiac myocyte cells demonstrated that a single beating cell can influence the rate of a neighbouring cell in close contact and that a group of heart cells in a culture, beating synchronously with a rapid rhythm, can act as pacemaker for a contiguous cell sheet [1]. Though the former results predicted that a rapidly beating region of tissue acts as pacemaker for a slower one and examined how the synchronization process of two isolated beating cardiac myocytes [2], the cell-to-cell connection could not be controlled completely without using microstructures on the cultivation plate. As means of attaining the spatial arrangement of cardiac myocytes, we have developed a new single-cell cultivation method and a system using agar microstructures, based on 1064-nm photo-thermal etching [3-6]. We have also developed the on-chip single-cell sorting method for cultivating particular cells chosen from clued mixture of cells [7], and have found the adaptation process of epigenetic memorization in cells by storing the information as the localization of proteins [8].

This paper reports the practical use of the agar chamber for screening the community size effect of the synchronization process of adjacent cardiac myocyte cells having independent oscillation.

**Figure 1:**
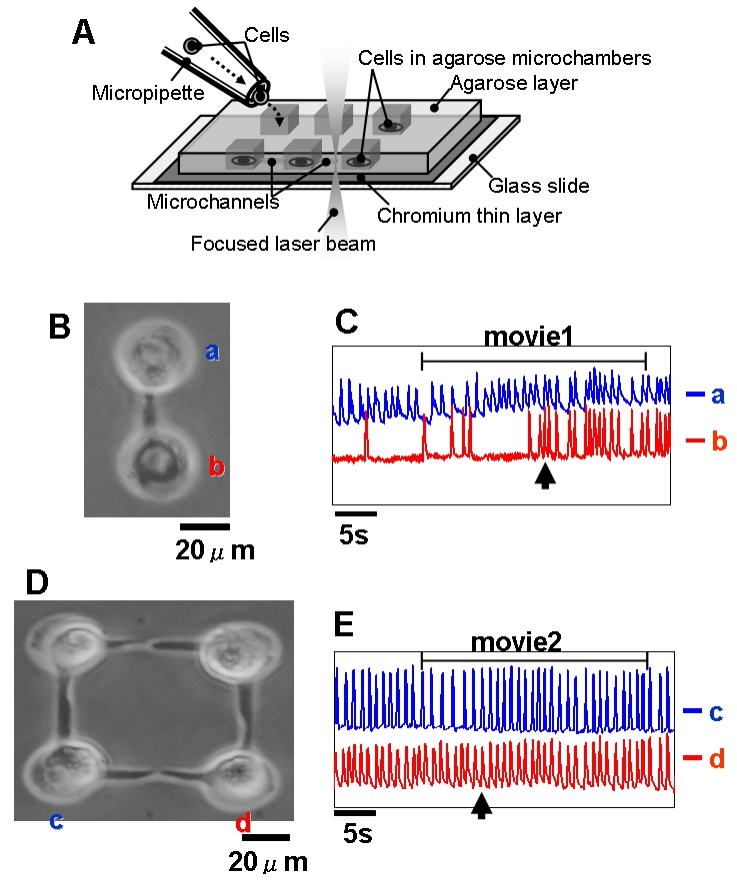
(A): Schematic drawing of the on-chip agar cultivation assay. (B): Optical micrograph of 24-h cultivation of two cardiac myocyte cells. (C): Time-course of oscillation of cardiac myocytes shown in Fig. (B). (D): Optical micrograph of 24-h cultivation of two sets of the synchronized pairs. (E): Time-course of oscillation of cardiac myocytes shown in Fig. (D).

Figure 1 shows the schematic drawing of the agar microchambers on the chip. The microchambers and microchannels were constructed by localized melting of a portion of the 5-μm-thick agar layer using a 1064-nm the infrared focused laser beam, a process we have termed photo-thermal etching. The 1064-nm laser beam is not absorbed by either water or the agar, and selectively melts a portion of the agar just near the chromium thin layer as this layer absorbs the beam energy. Microstructures such as holes and channels can be easily produced using this non-contact etching within only a few minutes without the requirement of any cast moulding process. The melting of agar by laser occurred as follows: (a) the 1064-nm infrared laser beam was focused on the agar layer on the glass slide; (b) the agar at the focal point and on the light pathway started to melt; (c) when the focused beam was moved parallel to the chip surface, a portion of agar around the focal spot of laser melted and diffused into water; (d) after the heated spot had been moved, a channel was created at the bottom of the agar layer connecting the two adjacent holes. The microscope confirmed the melting had occurred, and then either the heating was continued until the spot size reached the desired size, or the heating position was shifted to achieve the desired shape. Cardiac myocytes were cultivated in each hole of the agar microchambers on the chip as shown in Fig. 1. Collagen-type I (Nitta gelatin, Osaka, Japan) was coated on the glass layer surface to improve the attachment of the cell to the bottom of the microchambers.

Neonatal rat cardiac myocytes were isolated and purified as follows. First, the hearts of 1- to 3-day-old Wistar rats (Nippon Bio-supp. Center, Tokyo, Japan) were excised under ether anaesthesia. The ventricles were separated from the atria and then washed with phosphate buffered saline (PBS, 137 mM NaCl, 2.7 mM KCl, 8 mM Na_2 _HPO_4_, 1.5 mM KH_2_PO_4_, pH 7.4) containing 0.9 mM CaCl_2 _and 0.5 mM MgCl_2_. The ventricles were minced in PBS without CaCl_2 _or MgCl_2 _and then incubated in PBS containing 0.25% collagenase (Wako, Osaka, Japan) for 30 minutes at 37°C to digest the ventricular tissue. This procedure was repeated twice more and the cell suspension was then transferred to cell culture medium (DMEM [Invitrogen Corp., Carlsbad, CA USA] supplemented with 10% fetal bovine serum, 100 U/ml penicillin, and 100 μg/ml Streptomycin) at 4°C. The cells were filtered through a 40-μm nylon mesh and centrifuged at 180 g for 5 minutes at room temperature. The cell pellet was re-suspended in a HEPES buffer (20 mM HEPES, 110 mM NaCl, 1 mM NaH_2_PO_4_, 5 mM glucose, 5 mM KCl, and 1 mM MgSO_4_, pH 7.4). Cardiac myocytes present in the suspension were separated from other cells (i.e., fibroblasts and endothelial cells) by the density centrifugation method. The cell suspension was then layered onto 40.5% Percoll (Amersham Biosciences, Uppsala, Sweden) diluted in the HEPES buffer, which had previously been layered onto 58.5% Percoll diluted in the same buffer. The cell suspension was then centrifuged at 2200 g for 30 minutes at room temperature. Cardiac myocytes were retrieved from the interface of the 40.5% and 58.5% Percoll layers. Retrieved cells were then re-suspended in the cell culture medium. An aliquot (5- μl) of the suspension was diluted to achieve a final concentration of 3.0 × 10^5 ^cells/ml then plated into the chip. Individual cardiac myocytes were picked up by a micropipette and manually introduced into each chip microchamber and incubated on a cell-cultivation microscope system at 37°C in the presence of a humidified atmosphere of 95% air /5% CO_2_. It should be noted that because the microchamber sidewalls were made of agar, then the cells could not easily pass over the chambers.

Phase-contrast microscopy was used to measure the contraction rhythm of the cardiac myocytes and the network formation of cells in the two adjacent chambers that were connected by the focused beam.

The spontaneous contraction rhythm of cultured cardiac myocytes was evaluated by a video-image recording method. Images of beating cardiac myocytes were recorded with a CCD camera through the use of a phase contrast microscope. The sizes (cross-section of volume) of cardiac myocytes, which changed considerably with contraction, were also analyzed and recorded every 1/30 s by a personal computer with a video capture board.

Figure 1 shows a micrograph image of two isolated, independently beating cardiac myocytes coming into contact through the microchannel. Ninety min after the physical contact, the two connected cells started to oscillate synchronously. The time course change of the heart beating was as shown in Fig. 1. As shown in the graph, the process of synchronization was accomplished only after one of the cells stopped beating and then synchronized its oscillation with other cell. Movie 1 (see additional file 1 "movie1.mpg") depicts the process of beating synchronization. Once the synchronized oscillation of the two cells was accomplished (arrowhead in Fig. 1), then the two cells maintained synchronization similar to that observed in whole tissue. A time interval of approximately 90 min was needed to form the gap junction between the two adjacent cells.

The same method was also used to make more complicated network patterns of cardiac myocytes. Figure 1 shows a micrograph of a four-cell network. As shown in the graph (Fig. 1), two sets of the beating pairs synchronized without having to stop unlike that previously observed for the synchronization of isolated cells (see additional file 2 "movie2.mpg"). This suggests that the synchronization dynamics and rhythm of the cell group is more stable than that of single cells.

In conclusion, we present a 1064-nm photo-thermal etching technology with which to create agarose microchambers for growing networks of cardiac myocyte cells. Using the system, we first observed the differences of the synchronization process of cardiac myocyte cells and their dependence on community size. This system has great potential for use in the biological/medical fields for cultivating the next stage of single-cell based networks and measuring their properties in laboratories.

## Authors' contributions

KK and TK carried out the microchamber design, cell preparation, single cell cultivation and observation, image analysis. Both authors contributed equally to this article. KY conceived of the study, and participated in its design and coordination. All authors read and approved the final manuscript.

## Supplementary Material

Additional File 1 two cells. synchronized oscillation of the two cells Click here for file

Additional File 2 two sets of cells. synchronized oscillation of the two sets of cells Click here for file
